# Supplementary material for: Personalising intravenous to oral antibiotic switch decision making through fair interpretable machine learning
Source: Nat Commun. 2024 Jan 13;15:506. doi: 10.1038/s41467-024-44740-2 (PMC10787786; doi:10.1038/s41467-024-44740-2)
Supplement: Supplementary file 1 — Supplementary information [file 41467_2024_44740_MOESM1_ESM.pdf]

Supplementary Material

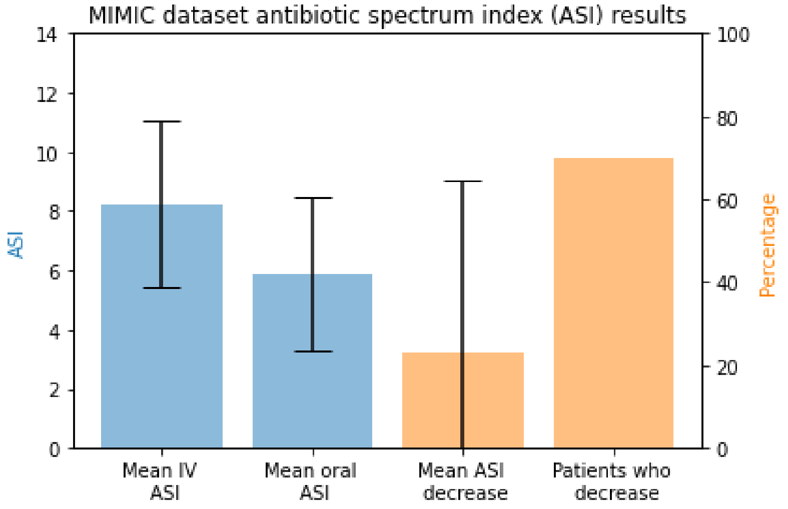

Figure 1: MIMIC antibiotic spectrum index results for the day before switching (IV) and the day after switching (oral). Error bars indicate the standard deviation.

ASI;Antibiotic spectrum index

Table 1: Feature sets identified by the genetic algorithm. The first 5 were used within the short model while all the features were used in the long model. The short feature set contained the 1st, 2nd, 4th, 5th and 6th SHAP ranked features, while the long feature set contained those same 5 alongside an additional 32. Details of the Catch22 transformation key can be found in Supplementary Table. 2.

Clinical parameter	Catch22 transformation key	Calculated over the entire current stay or the given day	Calculated as the difference from the previous day	SHAP value
Systolic blood pressure	17	Entire current stay	✗	2.27
Heart rate	17	Given day	✗	2.05
Mean blood pressure	11	Entire current stay	✗	1.62
O2 saturation pulseoxymetry	3	Entire current stay	✗	1.38
GCS motor response	15	Entire current stay	✗	1.37
Heart rate	17	Entire current stay	✗	1.27
GCS verbal response	10	Entire current stay	✗	1.16
Heart rate	14	Given day	✗	1.15
O2 saturation pulseoxymetry	17	Given day	✗	1.06
GCS motor response	10	Entire current stay	✗	0.98
Respiratory rate	17	Entire current stay	✗	0.92
Mean blood pressure	5	Entire current stay	✗	0.91
Respiratory rate	6	Entire current stay	✓	0.80
Temperature	7	Entire current stay	✓	0.77
Systolic blood pressure	15	Entire current stay	✗	0.77
Heart rate	9	Entire current stay	✓	0.77
Systolic blood pressure	5	Entire current stay	✓	0.75
Heart rate	3	Given day	✗	0.73
O2 saturation pulseoxymetry	9	Given day	✗	0.72
O2 saturation pulseoxymetry	2	Given day	✗	0.69
GCS motor response	23	Entire current stay	✗	0.69
GCS verbal response	7	Entire current stay	✗	0.68
GCS motor response	13	Given day	✗	0.63
Heart rate	16	Entire current stay	✗	0.63
GCS motor response	0	Entire current stay	✗	0.62
Temperature	10	Given day	✓	0.61
Heart rate	21	Given day	✗	0.61
GCS motor response	22	Given day	✗	0.58
Diastolic blood pressure	3	Entire current stay	✗	0.56
GCS eye opening	21	Given day	✗	0.55
Respiratory rate	12	Given day	✓	0.55
GCS motor response	8	Entire current stay	✗	0.52
O2 saturation pulseoxymetry	1	Entire current stay	✓	0.52
Temperature	7	Entire current stay	✗	0.52
Temperature	6	Entire current stay	✓	0.51
Respiratory rate	3	Given day	✗	0.51
Heart rate	8	Given day	✗	0.50

SHAP;SHapley Additive exPlanations, GCS;Glasgow Coma Scale

Table 2: Catch22 time-series transformation key. Note that the mean and standard deviation were also included. More information on these transformations can be found at `https://feature-based-time-series-analys.gitbook.io/catch22-features/`.

Key	Corresponding transformation
0	DN_HistogramMode_5
1	DN_HistogramMode_10
2	CO_f1ecac
3	CO_FirstMin_ac
4	CO_HistogramAMI_even_2_5
5	CO_trev_1_num
6	MD_hrv_classic_pnn40
7	SB_BinaryStats_mean_longstretch1
8	SB_TransitionMatrix_3ac_sumdiagcov
9	PD_PeriodicityWang_th0_01
10	CO_Embed2_Dist_tau_d_expfit_meandiff
11	IN_AutoMutualInfoStats_40_gaussian_fmmi
12	FC_LocalSimple_mean1_tauresrat
13	DN_OutlierInclude_p_001_mdrmd
14	DN_OutlierInclude_n_001_mdrmd
15	SP_Summaries_welch_rect_area_5_1
16	SB_BinaryStats_diff_longstretch0
17	SB_MotifThree_quantile_hh
18	SC_FluctAnal_2_rsrangefit_50_1_logi_prop_r1
19	SC_FluctAnal_2_dfa_50_1_2_logi_prop_r1
20	SP_Summaries_welch_rect_centroid
21	FC_LocalSimple_mean3_stderr
22	Mean
23	Standard deviation

Table 3: Models hyperparameters and optimisation options.

Hyperparameter	Short model	Long model	Optimisation options
Learning rate	0.001	0.001	0.1, 0.01, 0.001, 0.0001, 0.00001
Batch size	256	256	16, 32, 64, 128, 256, 512
Optimiser	Adam	Adam	Adam, RMSprop, SGD
Input dimension	5	37	-
Batch normalization	BatchNorm1d	BatchNorm1d	-
Number of hidden layers	2	3	1, 2, 3, 4, 5, 6, 7, 8, 9
Hidden dimension 1	512	64	2, 4, 8, 16, 32, 64, 128, 256, 512, 1024
Hidden dimension 2	16	512	2, 4, 8, 16, 32, 64, 128, 256, 512, 1024
Hidden dimension 3	-	256	2, 4, 8, 16, 32, 64, 128, 256, 512, 1024
Output dimension	1	1	-
Activation function	ReLU	ReLU	-
Dropout	0.4	0.5	0, 0.1, 0.2, 0.3, 0.4, 0.5

SGD;Stochastic gradient descent, ReLU;Rectified linear unit

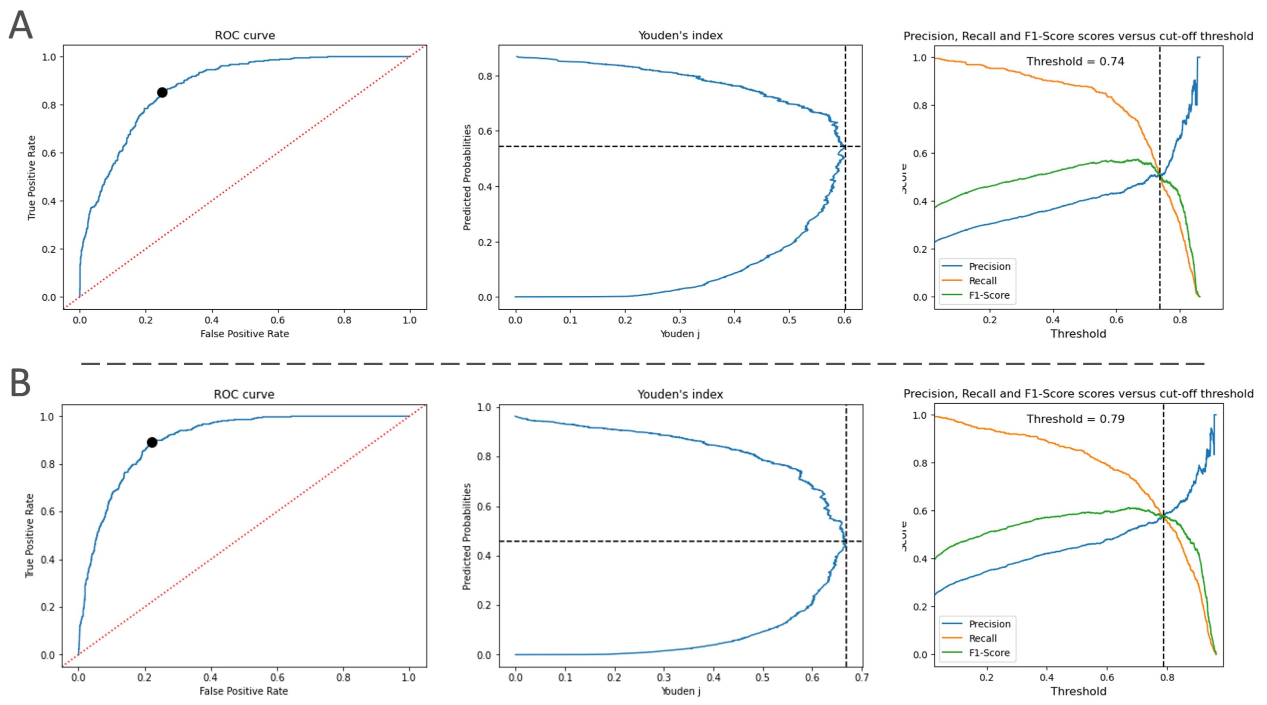

Figure 2: Preprocessing threshold optimisation graphs. ROC curve, Youden's index and Precision, Recall and F1-Score plots for the short model [A] (1st and 2nd threshold of 0.54 and 0.74 respectively) and the long model [B] (1st and 2nd threshold of 0.52 and 0.79 respectively).

ROC;Receiver operating characteristic

Table 4: Patient mean mortality by switch event temporal difference.

	Model	Short		Long	
	Threshold	1st	2nd	1st	2nd
Number of days between the real and predicted switch event	-7	0.00	0.00	0.00	0.00
	-6	0.50	0.00	0.00	0.00
	-5	0.00	0.50	0.40	0.00
	-4	0.17	0.00	0.00	0.33
	-3	0.43	0.17	0.00	0.00
	-2	0.11	0.38	0.09	0.17
	-1	0.04	0.29	0.00	0.00
	0	0.07	0.00	0.05	0.02
	1	0.00	0.00	0.00	0.05
	2	0.00	0.10	0.00	0.00
	3	0.00	0.00	0.00	0.00
	4	0.00	0.00	0.00	0.00
	5	0.00	0.00	0.00	0.00

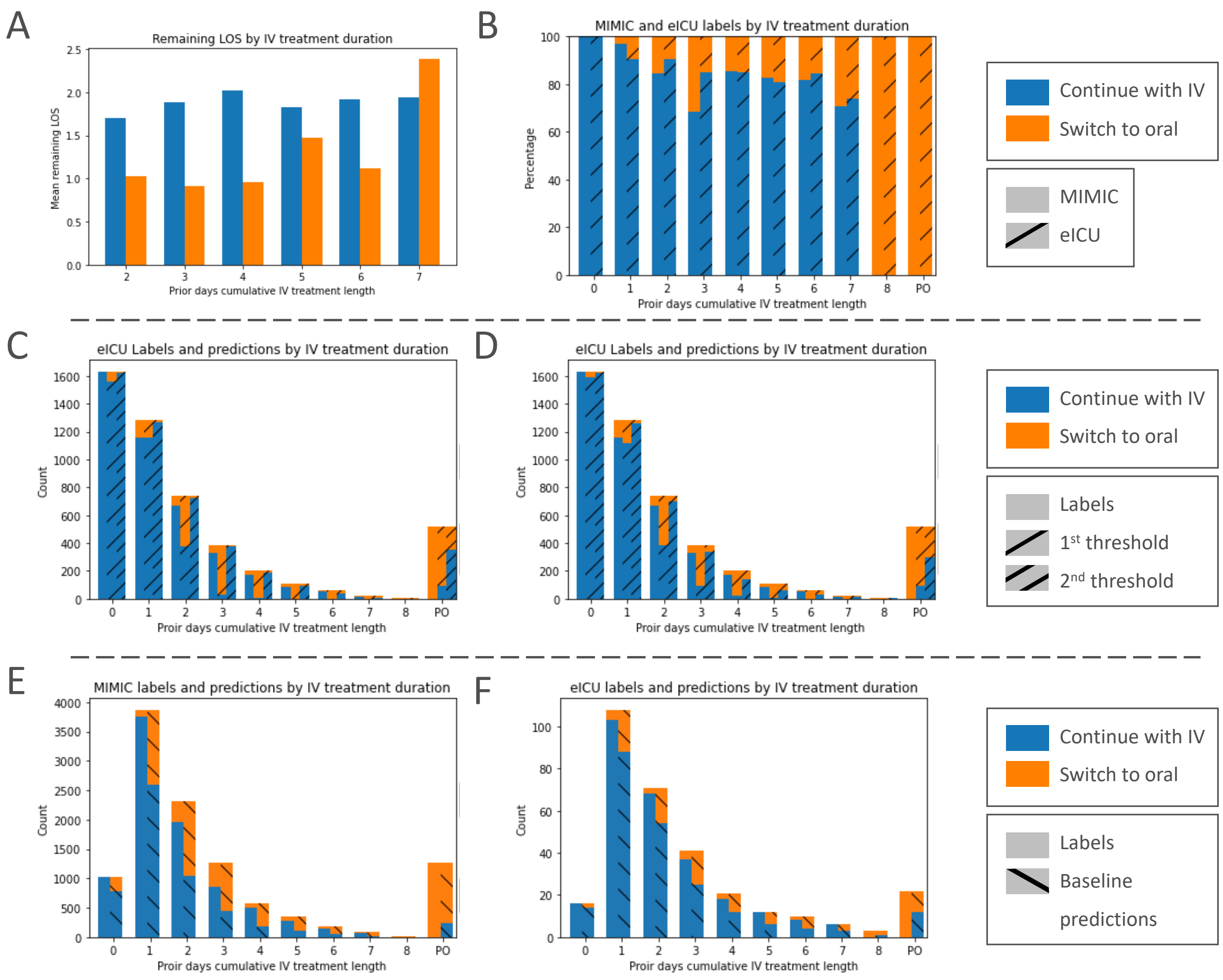

Figure 3: Labels, predictions, and LOS broken down by IV treatment duration. [A] demonstrates those patients on oral therapies on average have a shorter remaining LOS than those on IV. [B] shows switch labels for both the MIMIC and eICU datasets. They are generally consistent although eICU sees less patients switch on day 3. [C] and [D] are predictions for the transfer learning short and long models respectively on the eICU dataset. Models predict many more patients should not switch when compared to MIMIC results (Figure. 2). [E] and [F] show how baseline predictions compare to the labels for both MIMIC and eICU.

MIMIC;Medical Information Mart for Intensive Care, ICU;Intensive care unit, IV;Intravenous, LOS;Length of stay

Table 5: Threshold optimisation fairness results for the short model.

Sensitive attribute	Group	Original results AUROC	Original results TPR	Original results FPR	Original results EO	Threshold optimisation results AUROC	Threshold optimisation results TPR	Threshold optimisation results FPR	Threshold optimisation results EO
Age	20	0.73	0.74	0.27	✓	0.63	0.86	0.61	✗
	30	0.80	0.86	0.26	✓	0.72	0.73	0.28	✓
	40	0.78	0.81	0.25	✓	0.76	0.82	0.31	✓
	50	0.76	0.78	0.25	✓	0.81	0.86	0.25	✓
	60	0.79	0.82	0.23	✓	0.79	0.87	0.29	✓
	70	0.73	0.69	0.23	✓	0.78	0.87	0.31	✓
	80	0.77	0.81	0.26	✓	0.80	0.87	0.26	✓
	90	0.78	0.79	0.23	✗	0.78	0.86	0.3	✓
Race	Asian	0.79	0.83	0.24	✓	0.71	0.81	0.38	✓
	Black	0.78	0.83	0.27	✓	0.79	0.86	0.28	✓
	Hispanic	0.80	0.85	0.25	✓	0.76	0.84	0.31	✓
	Native	0.78	0.97	0.43	✗	0.75	0.93	0.43	✗
	Other	0.76	0.72	0.19	✓	0.78	0.84	0.29	✓
	Unknown	0.79	0.83	0.25	✓	0.81	0.86	0.23	✓
	White	0.77	0.79	0.24	✓	0.78	0.87	0.31	✓
Insurance	Medicaid	0.72	0.69	0.26	✗	0.74	0.82	0.34	✓
	Medicare	0.78	0.81	0.25	✓	0.77	0.88	0.33	✓
	Other	0.78	0.80	0.24	✓	0.79	0.9	0.33	✓

AUROC;Area under the receiver operating characteristic, TPR;True positive rate, FPR;False positive rate, EO;Equalised odds

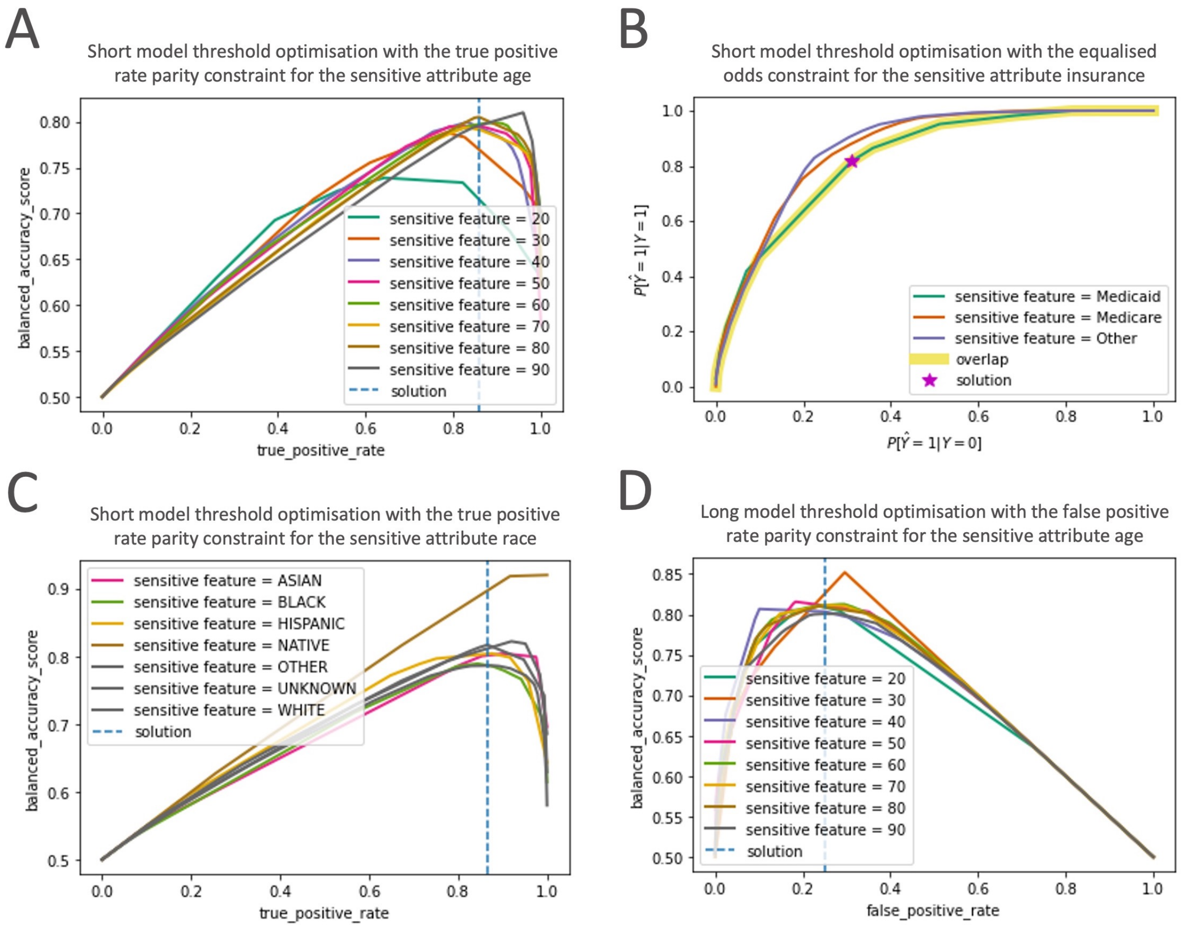

Figure 4: Sensitive attribute threshold optimisation graphs. [A] indicates that while fairness is now achieved by the short model for the 30 year old age group, 20 year old's suffer (Supplementary Table. 5). [B] demonstrates fair performance by the short model across insurance groups. [C] shows equalised odds cannot be achieved by the short model for the native population. [D] exhibits fair performance by the long model across age groups.

Table 6: Threshold optimisation fairness results for the long model.

		Origional results				Threshold optimisation results			
Sensitive attribute	Group	AUROC	TPR	FPR	EO	AUROC	TPR	FPR	EO
Age	20	0.76	0.77	0.24	✓	0.69	0.63	0.24	×
	30	0.74	0.64	0.20	×	0.78	0.88	0.31	✓
	40	0.77	0.8	0.26	✓	0.76	0.76	0.23	✓
	50	0.8	0.87	0.26	✓	0.81	0.88	0.25	✓
	60	0.8	0.84	0.24	✓	0.81	0.86	0.24	✓
	70	0.81	0.86	0.23	✓	0.81	0.88	0.25	✓
	80	0.81	0.85	0.23	✓	0.82	0.89	0.25	✓
	90	0.78	0.78	0.22	✓	0.78	0.81	0.25	✓

AUROC;Area under the receiver operating characteristic, TPR;True positive rate, FPR;False positive rate, EO;Equalised odds

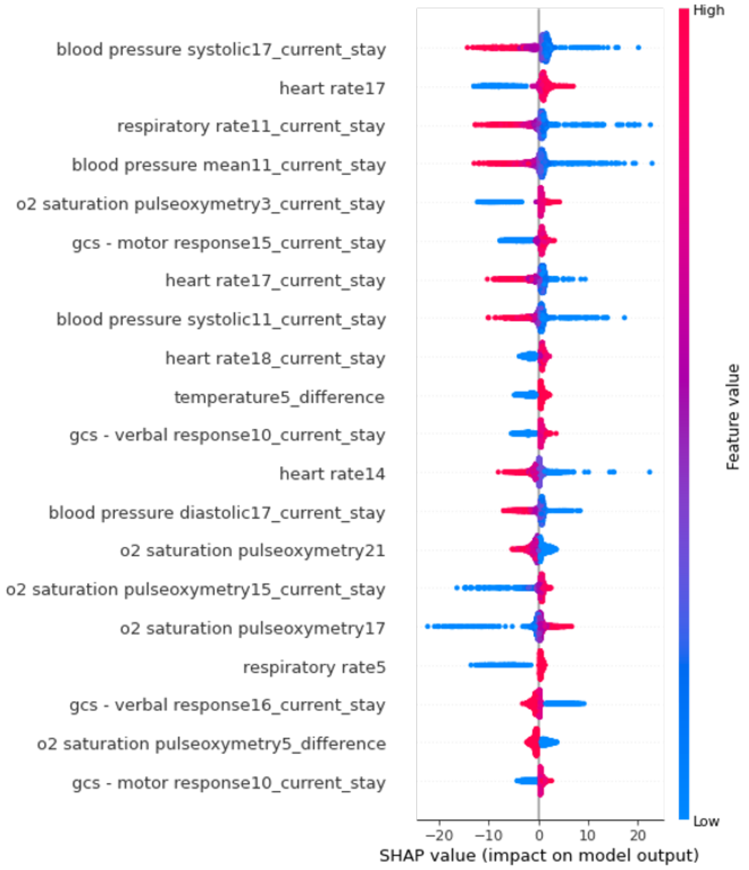

Figure 5: SHAP and feature value distribution for the 20 most important features. Notice how ‘respiratory rate11_current_stay’ and ‘blood pressure mean11_current_stay’ (features of importance 3 and 4 respectively) are very similar implying redundancy.

SHAP;SHapley Additive exPlanations

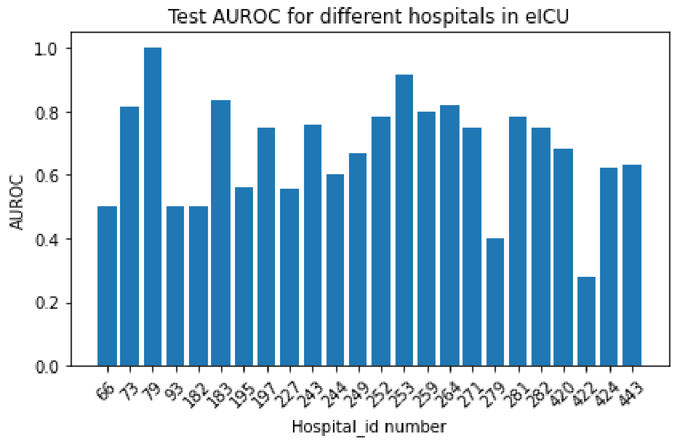

Figure 6: eICU test results broken down by hospitals.

ICU;Intensive care unit, AUROC;Area under the receiver operating characteristic

Table 7: Antibiotics selected for incomplete oral absorption analysis. * Selected due to nonlinear absorption.

Antibiotic	Oral bioavailability (%)
Amoxicillin	70
Ampicillin	50
Augmentin	70
Azithromycin	37
Cefpodoxime	50
Ciprofloxacin	70
Clarithromycin	50
Clindamycin*	90
DiCLOXacillin	49
Erythromycin	32
Flucloxacillin	50
Neomycin	3
Nitrofurantoin	80
Penicillin	60
Tetracycline	60
